# Response: Commentary: Vasopressin induced hyponatremia in infants <3 months of age in the neonatal intensive care unit

**DOI:** 10.3389/fped.2025.1571691

**Published:** 2025-04-10

**Authors:** Peter N. Johnson, Jamie L. Miller, Marjorie Makoni

**Affiliations:** ^1^ Department of Pharmacy, Clinical and Administrative Sciences, University of Oklahoma College of Pharmacy, Oklahoma City, OK, United States; ^2^ Section of Neonatology, Department of Pediatrics, University of Oklahoma College of Medicine, Oklahoma City, OK, United States

**Keywords:** vasopressin, hyponatremia, infant, neonate, NICU

A Commentary on Commentary: Vasopressin-induced hyponatremia in infants <3 months of age in the neonatal intensive care unit By Hebert A, Rios DR and McNamara PJ (2025). Front. Pediatr. 12:1514079. doi: 10.3389/fped.2024.1514079

We appreciate the letter to the Editor from Herbert colleagues (1) regarding our article entitled “Vasopressin induced hyponatremia in infants <3 months of age in the neonatal intensive care unit” (2). We agree with several of the concerns that the authors have noted about the use of vasopressin in neonates. However, we would like to take the opportunity to address several concerns outlined in their letter.

First, we have not noted any direct adverse clinical outcomes with hyponatremia due to vasopressin in our study. Due to the retrospective nature, we were limited in the number of outcomes we could assess and in the ability to account for all confounding variables. Unfortunately, we did not collect information on the presence of seizures since it was not our routine practice to monitor seizures with electroencephalography (EEG) on every baby receiving vasopressin. Therefore, it is not possible to extrapolate definitive data on seizures. However, this is a significant issue to address given the sequelae of hyponatremia and may be prudent to obtain an EEG on neonates if seizures are considered as a differential diagnosis in the case of refractory hypotension.

Second, the question was raised on how changes in practice may have impacted vasopressin-induced hyponatremia during the study period. Anecdotally, during the study period, we implemented an intentional increase in sodium supplementation alongside parenteral nutrition for neonates receiving vasopressin. This was achieved using our second lumen on central lines to deliver 0.9% sodium chloride and titrate per patients’ requirements. Additionally, our institution standardized the dilution of continuous intravenous fluid medications in 0.9% sodium chloride instead of dextrose-containing fluids wherever possible. However, we did not analyze the changes in the serum sodium during or post-vasopressin or the sodium supplementation provided prior and post-vasopressin therapy for each year of the study period. Since some time-periods had a small number of patients, it would be difficult to make significant conclusions, given the limited power and increased risk of a type II error. After completion of this study, the neonatal hemodynamics consult service was established, and they instituted targeted neonatal echocardiography, which allowed for personalized adjustments to vasopressin. In addition, they provided guidance on enhancing fluid and electrolyte management. It is our belief that these measures significantly reduced the severity of hyponatremia, and it is our hope to follow-up this study and determine the impact of this intervention on the incidence of hyponatremia with vasopressin.

Next, the question was raised about assessment of the relationship between the incidence of hyponatremia between the maximal and cumulative dose of vasopressin, sodium intake, and urinary sodium losses. During the study period, providers did not routinely order urinary sodium concentrations, so we are unable to comment further on this concern. In this study, we did collect the initial, peak, and final vasopressin dose (milliunits/kg/min) (Table 2), vasopressin duration (Table 2), and sodium intake from all sources (Table 4) (2). We also analyzed the sodium intake 24 h prior to nadir, day of nadir, and 24 h of nadir and only found a significant difference in the univariate analysis on the day of nadir and 24 h after nadir. These changes were likely a reaction of providers to the development of hyponatremia and is not surprising. We did employ a multiple logistic regression model and only included the duration of vasopressin since there was no difference in the initial, peak, and final vasopressin dose in our univariate analysis between groups. In addition, other studies have noted that only vasopressin duration has been associated with hyponatremia development rather than the dosages received (3).

Last, the authors inquired about corrective measures such as fluid restriction or sodium supplementation that were employed. We noted approximately one-third of patients with hyponatremia were fluid-restricted following development of hyponatremia (unpublished data). Table 4 in our article also further illustrates this point as there was a numerical but not statistical difference in reduction of fluid intake (ml/kg/day) in the hyponatremia group compared to 24 h prior to nadir and day of nadir (2). In addition, 41 (71.9%) of patients with hyponatremia received a 3% sodium chloride infusion to increase their serum sodium during the study period.

In summary, we appreciate the authors’ comments about our study. We agree that there remain several unanswered questions about the clinical outcomes of hyponatremia that occur in neonates receiving vasopressin. Given that we found for every 1 mEq/L increase in the baseline serum sodium concentration there was a decreased odds of hyponatremia by 10% (adjusted odds ratio 0.90; 95% CI: 0.83–0.99; *p* = 0.029), we hope that our study can raise awareness for providers to be proactive on initiation of sodium supplementation in patients with vasopressin infusions (2). Additionally, we hope that studies like our own can provide some guidance on the incidence, severity, and other potential risk factors for the development of hyponatremia with vasopressin.
